# Heparanase: A Potential New Factor Involved in the Renal Epithelial Mesenchymal Transition (EMT) Induced by Ischemia/Reperfusion (I/R) Injury

**DOI:** 10.1371/journal.pone.0160074

**Published:** 2016-07-28

**Authors:** Valentina Masola, Gianluigi Zaza, Giovanni Gambaro, Maurizio Onisto, Gloria Bellin, Gisella Vischini, Iyad Khamaysi, Ahmad Hassan, Shadi Hamoud, Omri Nativ, Samuel N. Heyman, Antonio Lupo, Israel Vlodavsky, Zaid Abassi

**Affiliations:** 1 Renal Unit, Department of Medicine, Verona, Italy; 2 Renal Unit, Columbus-Gemelli Hospital, Catholic University of the Sacred Heart, Roma, Italy; 3 University of Padova, Department of Biomedical Sciences Padova, Padova, Italy; 4 Gastroenterology, Rambam Health Care Campus, Haifa, Israel; 5 Internal Medicine A, Rambam Health Care Campus, Haifa, Israel; 6 Internal Medicine E, Rambam Health Care Campus, Haifa, Israel; 7 Department of Physiology and Biophysics, The Bruce Rappaport Faculty of Medicine, Technion, Haifa, Israel; 8 Department of Internal Medicine, Hadassah Medical Center, Jerusalem, Israel; 9 Cancer and Vascular Biology Research Center, Rappaport Faculty of Medicine, Technion, Haifa, Israel; 10 Research Unit, Rambam Health Care Campus, Haifa, Israel; University of Torino, ITALY

## Abstract

**Background:**

Ischemia/reperfusion (I/R) is an important cause of acute renal failure and delayed graft function, and it may induce chronic renal damage by activating epithelial to mesenchymal transition (EMT) of renal tubular cells. Heparanase (HPSE), an endoglycosidase that regulates FGF-2 and TGFβ-induced EMT, may have an important role. Therefore, aim of this study was to evaluate its role in the I/R-induced renal pro-fibrotic machinery by employing *in vitro* and *in vivo* models.

**Methods:**

Wild type (WT) and HPSE-silenced renal tubular cells were subjected to hypoxia and reoxygenation in the presence or absence of SST0001, an inhibitor of HPSE. *In vivo*, I/R injury was induced by bilateral clamping of renal arteries for 30 min in transgenic mice over-expressing HPSE (HPA-tg) and in their WT littermates. Mice were sacrificed 48 and 72 h after I/R. Gene and protein EMT markers (α-SMA, VIM and FN) were evaluated by bio-molecular and histological methodologies.

**Results:**

*In vitro*: hypoxia/reoxygenation (H/R) significantly increased the expression of EMT-markers in WT, but not in HPSE-silenced tubular cells. Notably, EMT was prevented in WT cells by SST0001 treatment. *In vivo*: I/R induced a remarkable up-regulation of EMT markers in HPA-tg mice after 48–72 h. Noteworthy, these effects were absent in WT animals.

**Conclusions:**

In conclusion, our results add new insights towards understanding the renal biological mechanisms activated by I/R and they demonstrate, for the first time, that HPSE is a pivotal factor involved in the onset and development of I/R-induced EMT. It is plausible that in future the inhibition of this endoglycosidase may represent a new therapeutic approach to minimize/prevent fibrosis and slow down chronic renal disease progression in native and transplanted kidneys.

## Introduction

Ischemia-reperfusion (I/R) injury is a relatively frequent clinical condition following several local and systemic events characterized by limited tissue perfusion such as delayed graft function, major vascular surgery complications, trauma and resuscitation, myocardial infarction, sickle cell disease and acute kidney injury (AKI) [1].

In particular, tissue hypoxia “*per-se*” and the consequent nutrient reduction cause alteration of normal cell metabolism/energy homeostasis [2]. As a result, ischemia activates cell death programs including apoptosis, necrosis and autophagy-associated death [3]. Additionally, the ischemic period is frequently associated with up-regulation of Toll-Like Receptors (TLRs) under the control of Hypoxia-inducible factors (HIFs) and NF-kB [4]. Following oxygen reperfusion, endogenous ligands from necrotic and apoptotic cells enhance the activation of innate and adaptive immune cells thus exacerbating the inflammatory tissue and organ injury [5].

As widely demonstrated in the kidney, the clinical consequences of I/R injury may be so severe to induce AKI and delayed graft function (DGF) in transplantation [6]. Both these events correlate with the development of tubulointerstitial fibrosis which impacts on long term function and could be responsible for the development of chronic allograft dysfunction (CAD) [7]. I/R injury affects mainly tubular and endothelial cells [6]. Hypoxia and hypoxia-generated reactive oxygen species (ROS) [8] together with pro-fibrotic cytokines and growth factors [9] activate in tubular cells several signaling programs that alter their physiology, thus sustaining the fibrotic process [10].

Epithelial-mesenchymal transition (EMT) of renal proximal tubular cells contributes directly to renal fibrosis [11]. Hypoxia [12] is one of the triggers for EMT such as FGF-2, TGF-β and ROS [13].

During the transdifferentiation process, renal tubular cells activate genetic programs which result in the loss of epithelial markers, up-regulation of mesenchymal markers such as vimentin (VIM), alpha-smooth muscle actin (α-SMA) and fibronectin (FN), loss of cell-cell and cell-basement membrane contact, and acquisition of a myofibroblast phenotype [12,14].

As demonstrated by our group, heparanase (HPSE) plays an important role in regulating renal EMT [15]. HPSE is an endoglycosidase that cleaves heparan sulfate (HS) chains and thus participates in extracellular remodeling [16]. Though HPSE is involved in a number of physiological processes (e.g., embryo implantation, hair growth, tissue repair and inflammatory processes) [16], special attention is paid to its involvement in pathological conditions including tumor progression and renal diseases. A role for HPSE has been identified in several proteinuric nephropathies [17–19] mainly in the pathogenesis of diabetic nephropathy (DN) [20–22] and in a model of septic AKI [23].

Recent studies have shown that HPSE is involved in the development of fibrosis in a DN mouse model [22]. Moreover, at the molecular level we have demonstrated that this enzyme may regulate FGF-2- and TGF-β-induced EMT of renal tubular cells [24,25] and that its inhibition prevents pro-fibrotic events [26].

Applying *in vitro* and *in vivo* models, bio-molecular and histological methodologies, the present study, focuses on the involvement of HPSE in hypoxia/reoxygenation (H/R)-induced EMT and its potential therapeutic relevance.

## Material and Methods

### Cell cultures and treatments

**Fig 1:**
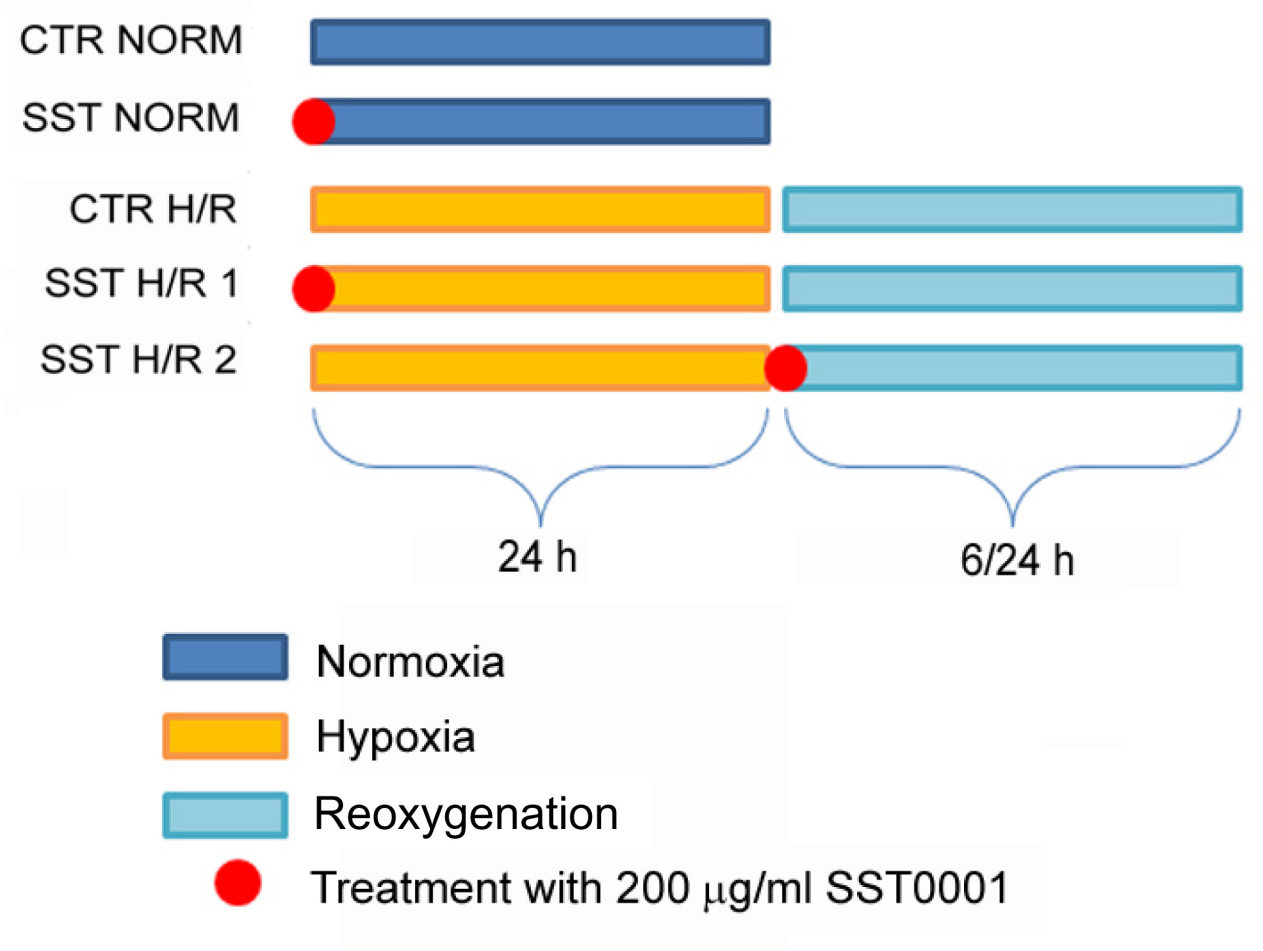
Schematic representation of *in vitro* experimental design. Serum starved HK-2 cells were exposed to 24 h of hypoxia and subsequently to reoxygenation (6 h for gene expression and 24 h for protein expression analysis). Cells were also treated or not with 200 μg/ml SST0001 (red balls) before the hypoxia (point #4; herein after referred to as H/R1) and before reoxygenation (point #5; herein after called H/R2). Control cells were cultured in normoxic conditions with or without SST0001 (points #1 and 2).

Human renal proximal tubular cell line HK2 (human kidney 2) cells were purchased from ATCC (CRL-2190^™^). The cells were grown in DMEM-F12 (EuroClone) (17.5 mM glucose) supplemented with 10% fetal bovine serum (Biochrom AG), 2 mM L-glutamine, penicillin (100 U/ml) and streptomycin (100 μg/ml). Cells were cultured at 37°C in a 5% CO_2_ water-saturated atmosphere to subconfluence and starved for 24 h in serum-free medium. A stably HPSE-silenced HK2 cell line was obtained by transfection with shRNA plasmid targeting human HPSE (NM_006665) purchased from OriGene, as previously described [21]. HPSE-silenced HK2 cells were grown in the same medium of wild-type (WT) HK2 cells supplemented with 0.75 μg/ml puromycin. Hypoxic condition was established for 24 h by using an Anaerob Atmosphere Generation Bags (68061 Sigma Aldrich) and an indicator test for checking the anaerobic conditions (59886 Sigma Aldrich). The system reduces oxygen concentration below 1% in 1 h without changing the medium pH. Subsequently, the cells were cultured under normoxic conditions for 6 h (gene expression analysis) or 24 h (protein expression analysis) (reoxygenation phase) [26]. The cells investigated after a complete cycle of H/R are hereinafter indicated as H/R cells. Control cells were grown in normoxic conditions for the same time periods. WT HK2 cells were also treated with or without 200 μg/ml SST0001 (Sigma-Tau Research Switzerland SA, Mendrisio, CH) both before hypoxia and prior to reoxygenation to mimic a possible prevention or pharmacological strategy (Fig 1). SST0001 is a modified non anti-coagulant heparin that is 100% N-acetylated and 25% glycol split [27]. Additionally, SST0001 acts by competing with physiologic heparin for heparanase catalityc site (and allosteric sites) with a reversible interaction [28,29].

### Gene expression analysis

Total RNA was extracted from cell monolayers and from frozen renal tissues using the “Trizol” reagent (Invitrogen), according to the manufacturer’s instruction. Yield and purity were checked using Nanodrop (EuroClone) and total RNA from each sample was reverse transcribed into cDNA using SuperScript II Reverse Transcriptase (Invitrogen). Real-time PCR was performed on an ABI-Prism 7500 using Power SYBR Green Master Mix 2X (Applied Biosystems). The comparative Ct method (ΔΔCt) was used to quantify gene expression and the relative quantification was calculated as 2^-ΔΔCt^. The presence of non-specific amplification products was excluded by melting curves analysis. The primers are listed in Table 1.

**Table 1:** Primers sequence list.

Gene	Forward 5’-3’	Reverse 5’-3’
**Human**		
GAPDH	ACACCCACTCCTCCACCTTT	TCCACCACCCTGTTGCTGTA
HPSE	ATTTGAATGGACGGACTGC	GTTTCTCCTAACCAGACCTTC
α-SMA	GAAGAAGAGGACAGCACTG	TCCCATTCCCACCATCAC
FN	GTGTGTTGGGAATGGTCGTG	GACGCTTGTGGAATGTGTCG
**Mouse**		
GAPDH	GGCAAATTCAACGGCACAGT	GTCTCGCTCCTGGAAGATGG
HPSE	CAAGAACAGCACCTACTCAAG	AGCAGTAGTCAAGGAGAAGC
α-SMA	TGCTGGACTCTGGAGATGGT	ACGAAGGAATAGCCACGCTC
VIM	TCCAGAGAGAGGAAGCCGAA	AAGGTCAAGACGTGCCAGAG

### Western Blotting

Cells from cultures were lysed in RIPA buffer for HIF-1 alpha investigation and in 50 mM Tris-HCl, pH 5.0, 150 mM NaCl,0.5% Triton X-100 with Complete Protease Inhibitor Mixture (Roche Applied Science) for the other cytoplasmic protein analyses. Briefly, equal amounts of proteins were treated in reducing sample buffer and denatured for 10 min at 100°C. Protein samples were then resolved in 10% SDS-PAGE and electrotransferred to nitrocellulose membranes. Non-specific binding was blocked for 1 h at room temperature with non-fat milk (5%) in TBST buffer (50 mM Tris-HCl, pH 7.4, 150 mM NaCl, 0.1% Tween 20). Membranes were exposed to primary antibodies directed against GAPDH (Santa Cruz sc-25778), FN (Santa Cruz sc-9068), α-SMA (Sigma A5228), HPSE (InSight HP130) or HIF-1 alpha (BD 610959), overnight at 4°C and incubated with a secondary peroxidase-conjugated antibody for 1 h at room temperature. The signal was detected with SuperSignals West Pico Chemiluminescent substrate solution (Pierce) according to the manufacturer’s instructions.

### Immunofluorescence

Sections (4 μm) were cut from paraffin embedded tissue and non-specific binding sites were saturated for 1 h at 37°C with PBS, 5% BSA. Sections were incubated at 4°C overnight with primary antibodies diluted in PBS supplemented with 1% BSA. The primary antibodies used were directed against VIM (Santa Cruz sc-7557), FN (Santa Cruz sc-9068), α-SMA (Sigma A5228), or HPSE (Santa Cruz sc-25826). Primary antibodies were revealed with an anti-goat-FITC for VIM, anti-rabbit-Cy3 for FN and HPSE, and anti-mouse-TR for α-SMA by incubation at room temperature for 45 min. Cell nuclei were visualized by Hoechst 33258. Images were obtained with a confocal LeicaSP5 microscope. Image processing was done with Image J (https://imagej.nih.gov/ij/).

### Animal model and induction of renal ischemia

Acute ischemia was induced in WT and heparanase-overexpressing (HPA-tg) [30] Balb/c mice. The investigation was conducted according to the guidelines of the Animal Use and Care Committee, Technion (Haifa), and according to the Guide for the Care and Use of Laboratory Animals (NIH Publication no. 85–23, 1996) as approved by the local committee for supervision of animal experiments. The Bruce Rappaport Faculty of Medicine, Technion (Haifa, Israel) Animal Care and Use Committee (IACUC) specifically approved this study.

WT and HPA-tg mice were anesthetized with sodium pentobarbital (50 mg/kg, IP) and placed on a controlled heating (thermoregulated) table, keeping the body temperature at 37°C. Both right and left renal arteries were exposed and clamped for 30 min during which time the kidney was kept warm and moist. The clamp was then removed, the kidney was observed for return of blood flow, and the abdominal wall incision was sutured. The mice were allowed to recover in a warmed cage for 48 and 72 h and blood samples were obtained at each time point for measurement of creatinine. All the mice were sacrificed by CO2 inhalation followed by cervical dislocation. The kidneys were harvested and weighted. One half of the left kidney was snap-frozen in liquid nitrogen and stored at −70°C until further molecular processing; the other half was fixed in formalin, paraffin-embedded, and sectioned (4 μm). Paraffin sections were used for PAS staining. Sham operated mice that underwent an identical procedure except for renal artery clamping, served as controls.

### Statistical Analysis

Mean ± S.D. of the real-time PCR data were calculated with Rest2009 software. Differences between normoxia and hypoxia or treated and untreated cells were compared using Two-tailed Student's t-test. A p-value < 0.05 was set as the level of significance for all tests. Gene expression differences in mouse samples were analyzed by linear regression models with group (WT-sham, WT-I/R 48h, TG-sham, TG-I/R 48h) entered as a categorical variable. Bonferroni-corrected adjusted means and differences were computed using the WT-sham as the referent group. A Bonferroni-corrected p-value < 0.05 was considered as statistically significant

## Results

### Hypoxia/Reoxygenation (H/R) increased HPSE expression in renal tubular epithelial cells

To identify whether H/R was able to modulate HPSE expression in renal tubular epithelial cells, we used wild type, HPSE-silenced and WT SST0001-treated HK2 cells. SST0001 is a specific inhibitor of HPSE.

**Fig 2:**
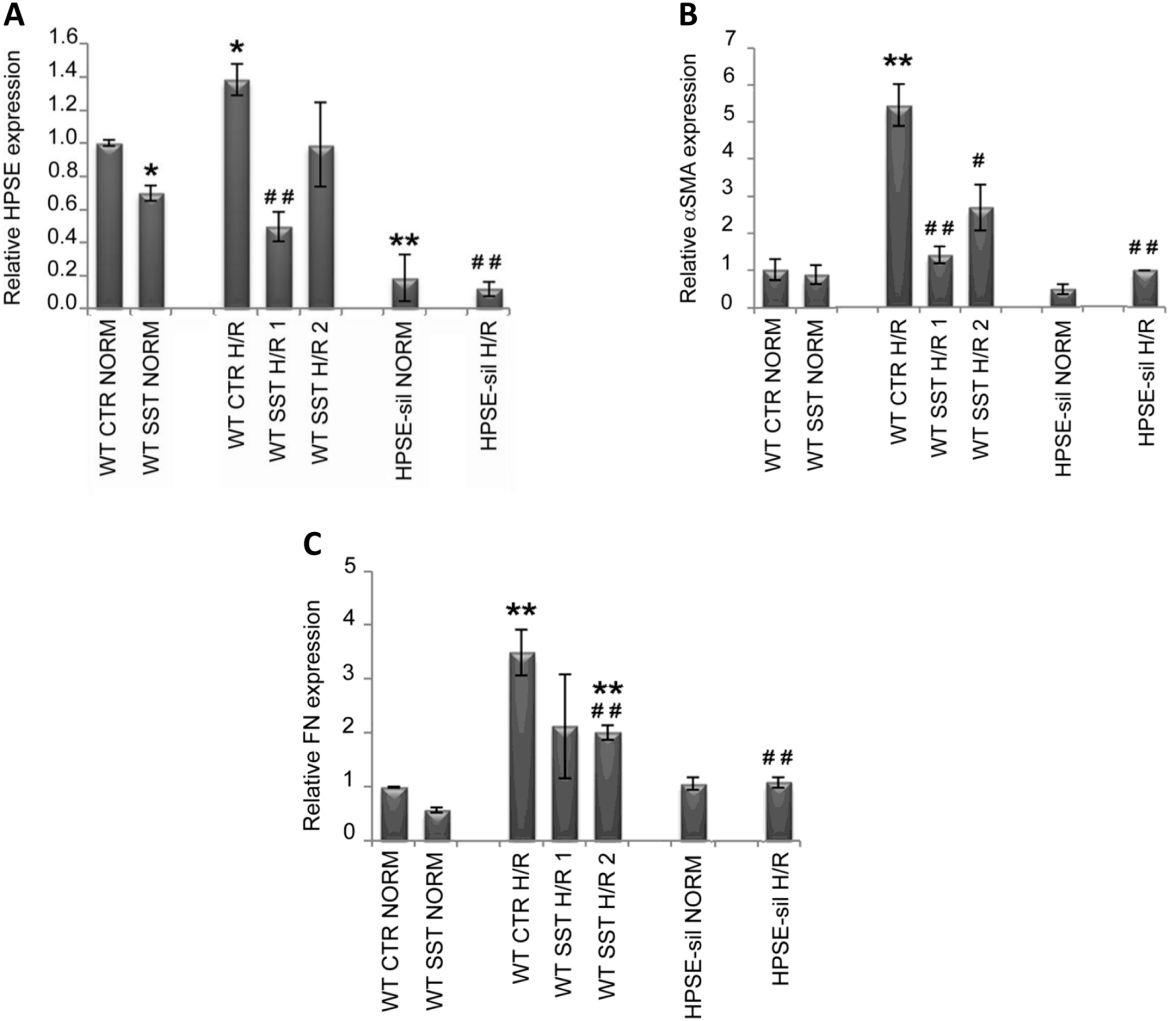
Heparanase (HPSE) and epithelial to mesenchymal transition (EMT)-associated gene expression in tubular cells exposed to hypoxia and reoxygenation (H/R). Wild type (WT) and Heparanase-silenced (HPSE-sil) HK2 cells exposed to hypoxia and reoxygenation (H/R); WT HK-2 cells were also treated or not with 200 μg/ml SST0001. Gene expression analysis of **A)** Heparanase (HPSE), **B)** alpha smooth muscle Actin **(**α-SMA) and **C)** Fibronectin (FN) evaluated by real-time PCR. Data were normalized to GAPDH expression. NORM = normoxia. Mean ± S.D (error bars) of two separate experiments performed in triplicate. **p<0.001, *p<0.05 vs. WT CTR NORM; ##p<0.001, # p<0.05 vs WT CTR H/R.

**Fig 3:**
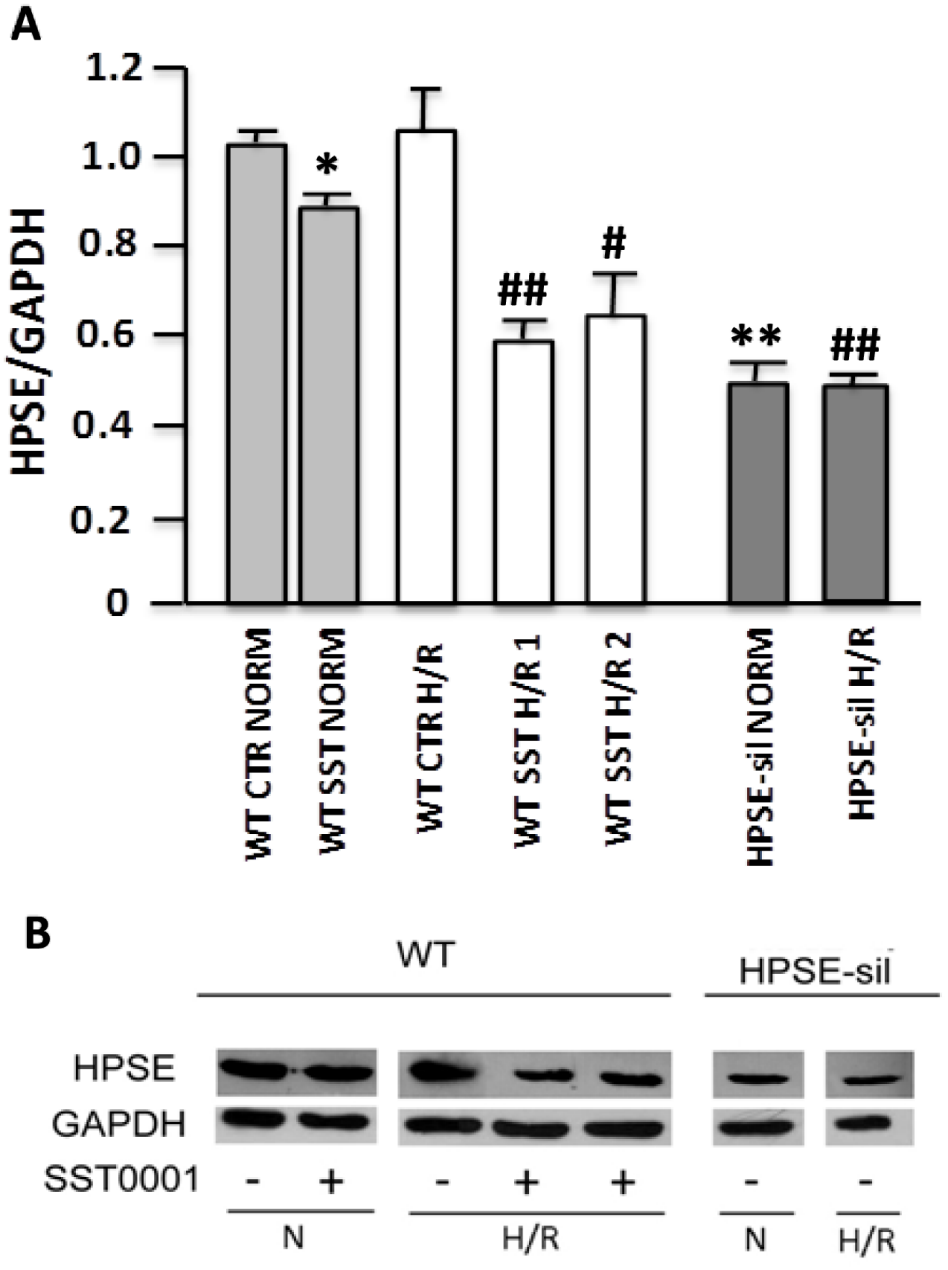
Heparanase (HPSE) protein expression in tubular cells exposed to hypoxia and reoxygenation (H/R). Wild type (WT) and Heparanase-silenced (HPSE-sil) HK2 cells exposed to hypoxia and reoxygenation (H/R); WT HK-2 cells were also treated or not with 200 μg/ml SST0001. **A)** Quantification of HPSE protein levels assessed by Western blot. GAPDH was included as loading control. NORM = normoxia. **B)** Representative image of western blot experiments. **p<0.001, *p<0.05 vs. WT CTR NORM; ##p<0.001, # p<0.05 vs WT CTR H/R.

Interestingly, as showed in Fig 2A gene expression analysis revealed that H/R was able to induce a significant *HPSE* up-regulation in WT cells, but neither in HPSE-silenced cells nor in those treated with the specific inhibitor. At protein level (Fig 3A), we found a slight, but not statistically significant increment of this enzyme after H/R. Notably, treatment of WT cells with SST0001 reduced HPSE gene expression below basal levels.

These data suggest that HPSE is modulated by H/R and it could be involved in the complex biological machinery associated with this event.

### HPSE-silencing inhibits EMT induced by H/R in renal tubular epithelial cells

Based on previous literature evidences suggesting a central role of HPSE in the EMT of renal tubular epithelial cells [15], we decided to measure its contribution in the profibrotic machinery induced by H/R.

To this purpose, major mesenchymal markers of EMT were measured after H/R in wild type HK2 cells and those silenced for HPSE or treated with the inhibitor SST0001.

**Fig 4:**
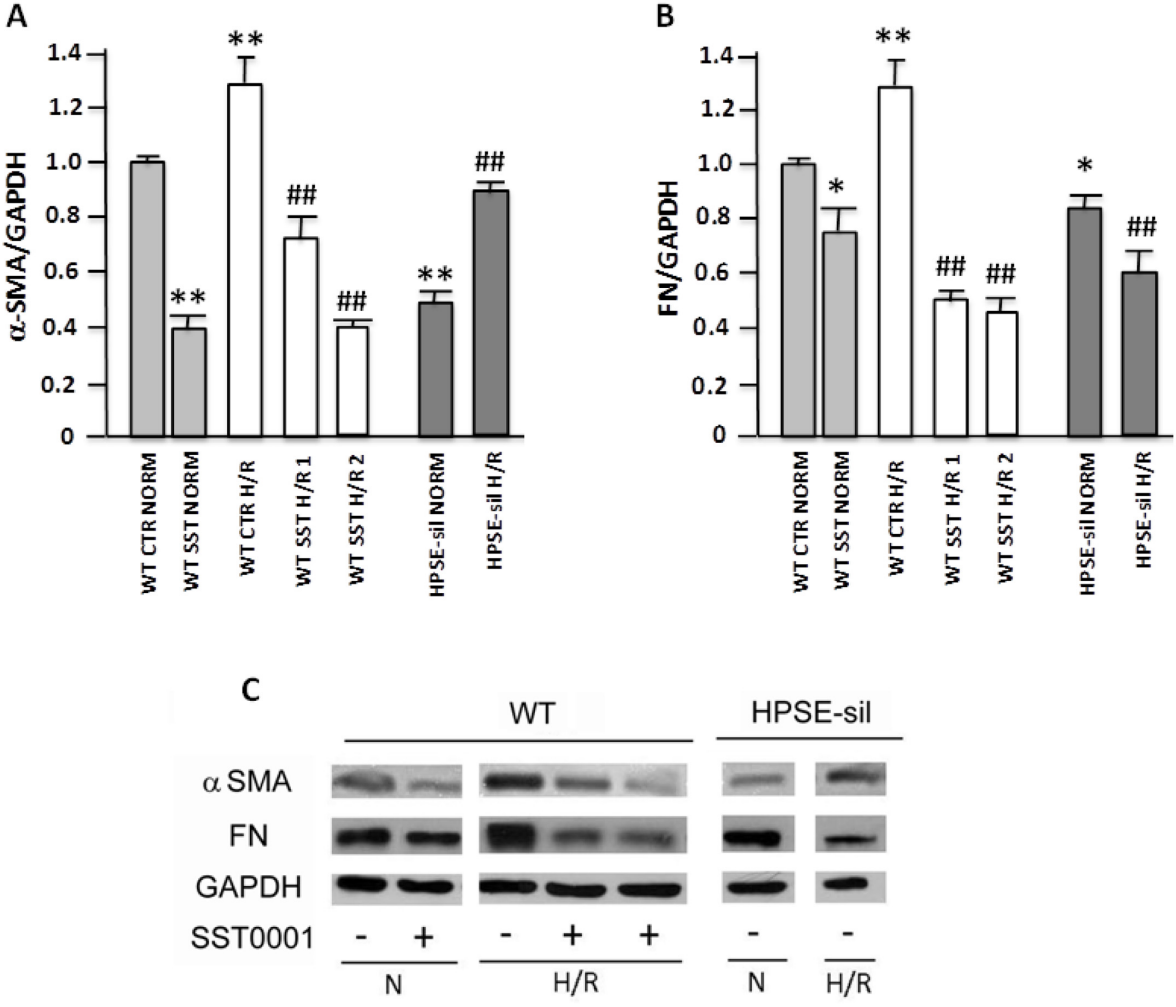
Epithelial to mesenchymal transition (EMT)-associated protein expression in tubular cells exposed to hypoxia and reoxygenation (H/R). Wild type (WT) and Heparanase-silenced (HPSE-sil) HK2 cells exposed to hypoxia and reoxygenation (H/R); WT HK-2 cells were also treated or not with 200 μg/ml SST0001. **A)** alpha smooth muscle Actin **(**α-SMA) and **B)** Fibronectin (FN) protein levels quantification assessed by western blot analysis. GAPDH was included as loading control. NORM = normoxia. **C)** Representative image of western blot experiments. **p<0.001, *p<0.05 vs. WT CTR NORM; # #p<0.001 vs. WT CTR H/R. **p<0.001, *p<0.05 vs. WT CTR NORM; ##p<0.001, # p<0.05 vs WT CTR H/R.

**Fig 5:**
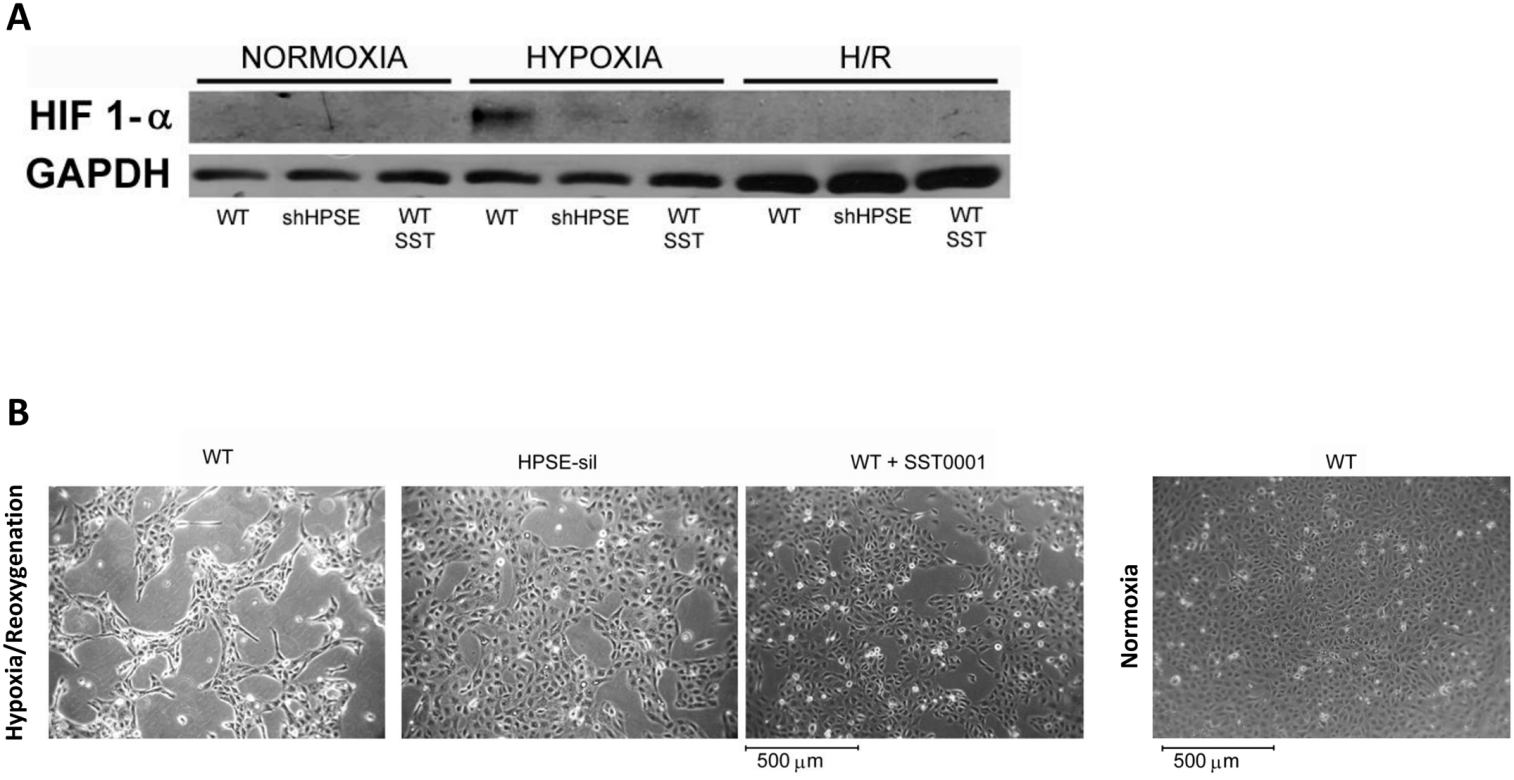
Morphological changes in tubular cells exposed to hypoxia and reoxygenation (H/R) and Hypoxia-inducible factor 1-alpha (HIF-1α) expression. Wild type (WT) and Heparanase-silenced (HPSE-sil) HK2 cells exposed to hypoxia and reoxygenation (H/R); WT HK-2 cells were also treated or not with 200 μg/ml SST0001. **A)** HIF-1α protein levels assessed by Western blot analysis. GAPDH was included as loading control. NORM: Normoxia. Representative image of western blot experiments. **B)** Representative images of WT cells treated or untreated with SST0001 and HPSE-silenced HK2 cells grown on a glass surface. Cells were exposed to normoxia or 24 h hypoxia followed by 24 h reoxygenation.

Gene and protein expression analyses revealed that H/R up-regulates the production of both α-SMA and FN in WT tubular cells. Treatment with SST0001 significantly reduced H/R-associated EMT, while HPSE-silenced cells exposed to H/R showed low expression level of both EMT markers (Figs 2B, 2C and 4). Our results confirmed that protein levels of HIF-1α, the master regulator of cell response to hypoxia, was significantly increased after hypoxia in WT renal tubular cells. As previously reported, 24 hours after reperfusion HIF-1α levels returned to baseline [31] (Fig 5A).

This effect was not present in cells silenced for HPSE or treated with SST0001 (Fig 5A).

Additionally, optical microscopy revealed that only wild type cells showed a morphological change after H/R displaying an elongated phenotype (Fig 5B).

### Ischemia and Reperfusion (I/R) caused HPSE up-regulation also in animal model

To assess whether I/R was able to induce up-regulation of HPSE *in vivo*, we used a wild-type (WT) mouse model in which I/R injury was induced by bilateral clamping of renal arteries for 30 min. Mice were sacrificed 48 and 72 h after I/R.

**Fig 6:**
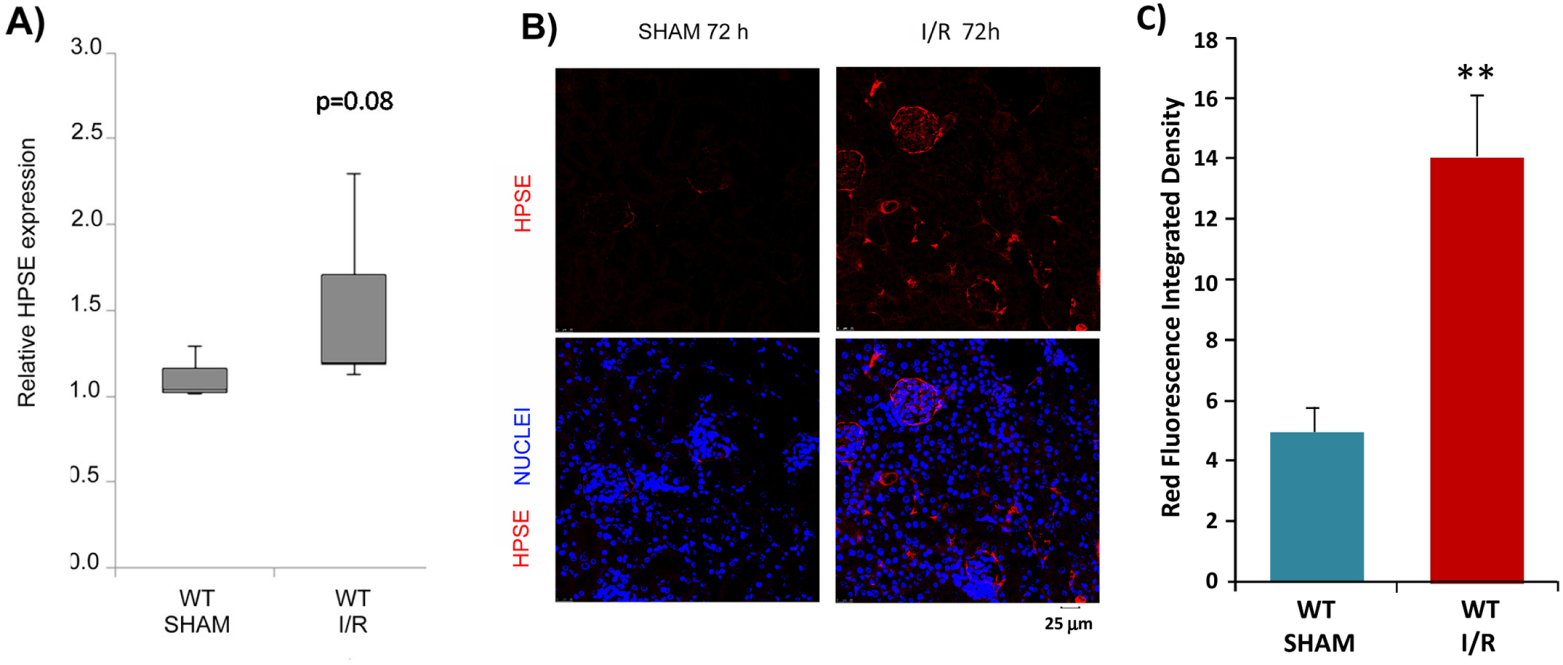
*In vivo* Heparanase (HPSE) expression induced by Ischemia and Reperfusion (I/R) kidney injury. **A)** Box plot representing relative gene expression of HPSE evaluated by real-time PCR in renal tissue extract from Wild type (WT) mice. Results were normalized to GAPDH expression. Mean ± S.E (error bars) of two separate experiments performed in triplicate. **B)** Representative immunofluorescence staining for HPSE in cortical renal tissues of WT mice 72 h after sham operation or I/R kidney injury. I/R, ischemia/reperfusion. **C)** Quantification of the immunofluorescence staining. **p<0.001vs. WT SHAM.

Interestingly, WT mice after 48 hours of reperfusion showed an increment of HPSE gene expression in renal tissue (Fig 6A). However, this did not reach the statistical significance (p = 0.08) probably because the low number of experiments.

Immunofluorescence staining of renal tissue 72 hours post-reperfusion confirmed the up-regulation of HPSE at both glomerular and tubulointerstitial levels (Fig 6B).

### Mice over-expressing HPSE activate EMT

To assess whether an up-regulation of HPSE was able to predispose to activation of EMT program, we used transgenic mice over-expressing HPSE (HPA-tg) and their WT littermates undergoing I/R injury by bilateral clamping of renal arteries for 30 min. Also in this case, mice were sacrificed 48 and 72 h after I/R.

Interestingly, gene expression analysis of total kidney lysates 48 hours after I/R and immunofluorescence staining for α-SMA and VIM 72 hours post-reperfusion showed that HPA-tg mice exhibited remarkable up-regulation of these biological elements.

**Fig 7:**
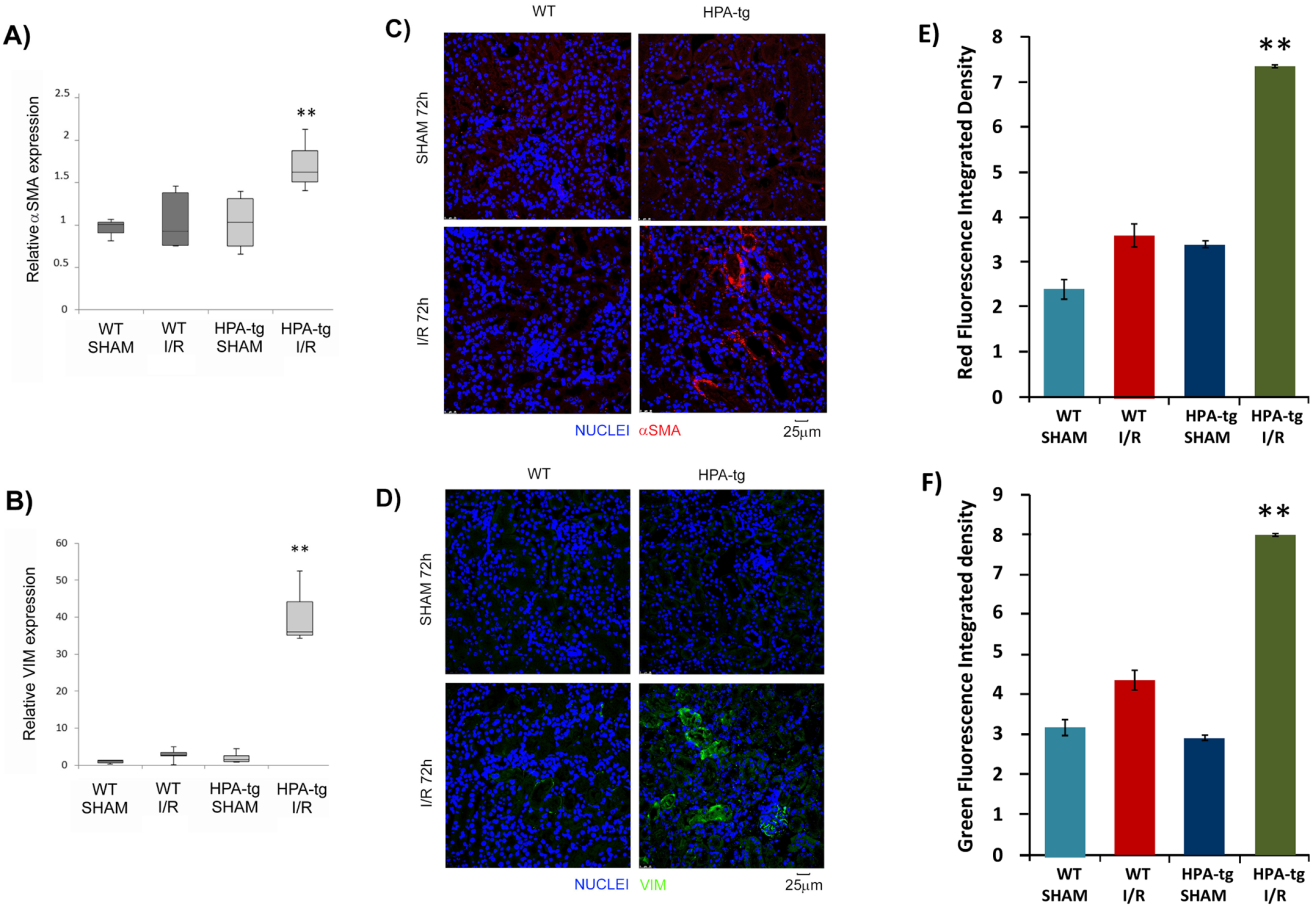
Epithelial to mesenchymal transition (EMT) markers in control (SHAM) mice vs. mice after Ischemia and Reperfusion (I/R) kidney injury. Relative gene expression of **A)** alpha smooth muscle Actin **(**α-SMA) and **B)** Vimentin (VIM) evaluated by real-time PCR in renal tissue extracts from WT and HPA-tg mice subjected to I/R kidney injury. Mean ± S.E (error bars) of two separate experiments performed in triplicate. **p<0.001 vs WT SHAM. Representative immunofluorescence staining for **C)** α-SMA and **D)** VIM in renal tissue of WT and HPA-tg mice subjected to I/R. **E)** Quantification of the immunofluorescence staining. **p<0.001vs. WT HPA-tg SHAM.

Instead, although we found an HPSE over-expression after I/R (Fig 6A and 6B), WT mice showed only a slight but not statistically significant increment of the transcriptional level of EMT markers after 48 hours and no increment in protein expression 72 hours after I/R (Fig 7). This could be explained by the higher complexity of the *in vivo* EMT and pro-fibrotic biological machinery compared to the *in vitro* one.

In addition, since I/R is accompanied also by the activation of inflammatory response we observed an increased macrophages infiltration in HPA-tg mice respect to wt mice 72 hours after I/R (S1 Fig).

### HPA-tg animals were more susceptible to renal function impairment compared to wild type

To measure whether HPA-tg animals were more predisposed to develop renal function impairment after I/R compared to WT, we measured serum-creatinine (SCr) levels in both mice model 72 hours after this insult.

**Fig 8:**
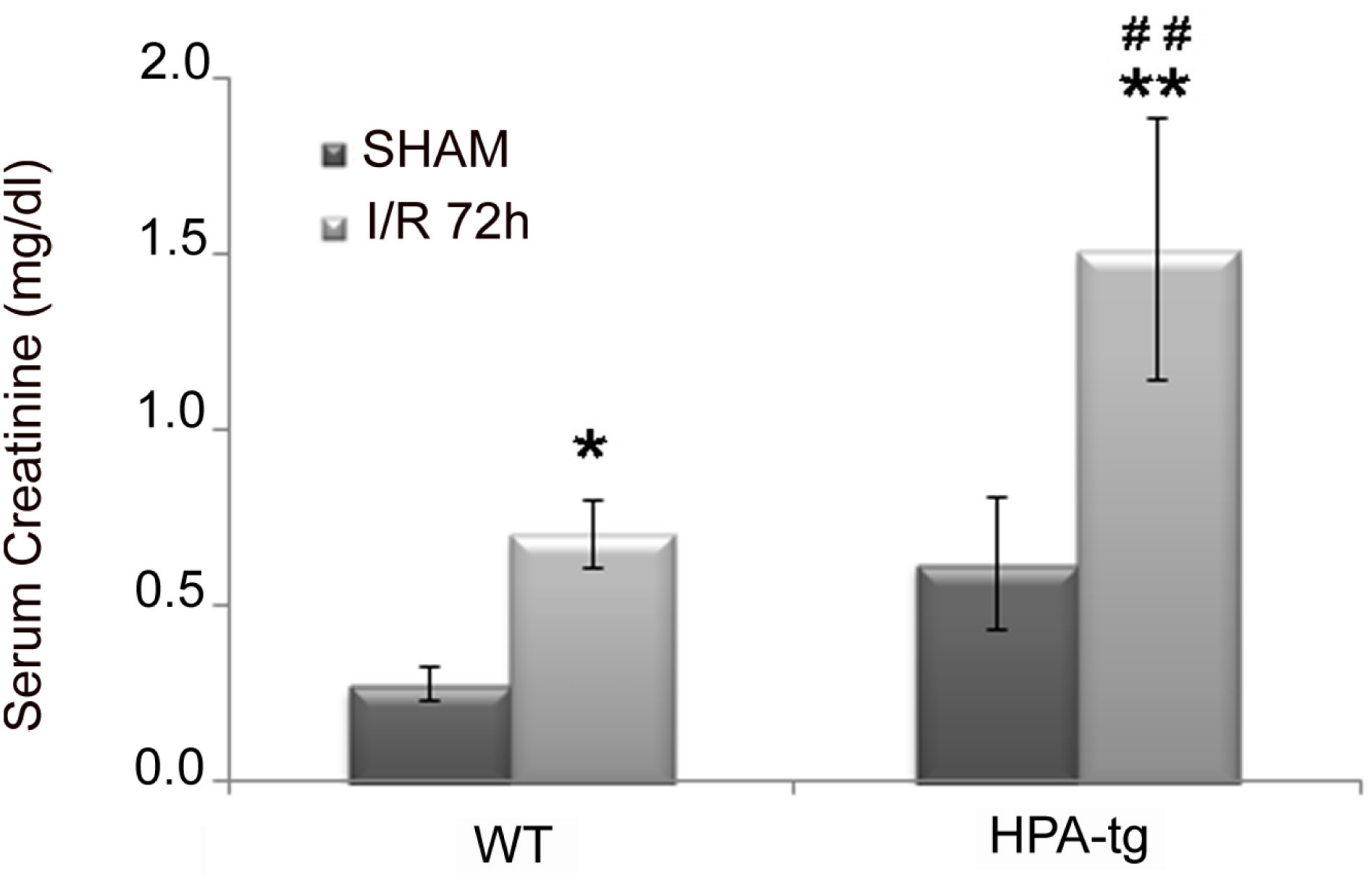
Renal function in Wild Type (WT) and HPA-tg mice subjected to Ischemia and Reperfusion (I/R). Effects of Acute kidney Injury (AKI) on serum creatinine (SCr) after I/R insult in WT and HPA-Tg mice. Results are expressed as Mean±SEM. *p<0.05, **p<0.001 vs. SHAM; ##p<0.001vs. WT I/R 72 h.

Interestingly, although SCr was higher in both WT and HPA-tg mice after I/R, this effect was greater in HPA-tg mice compared to controls (Fig 8).

**Fig 9:**
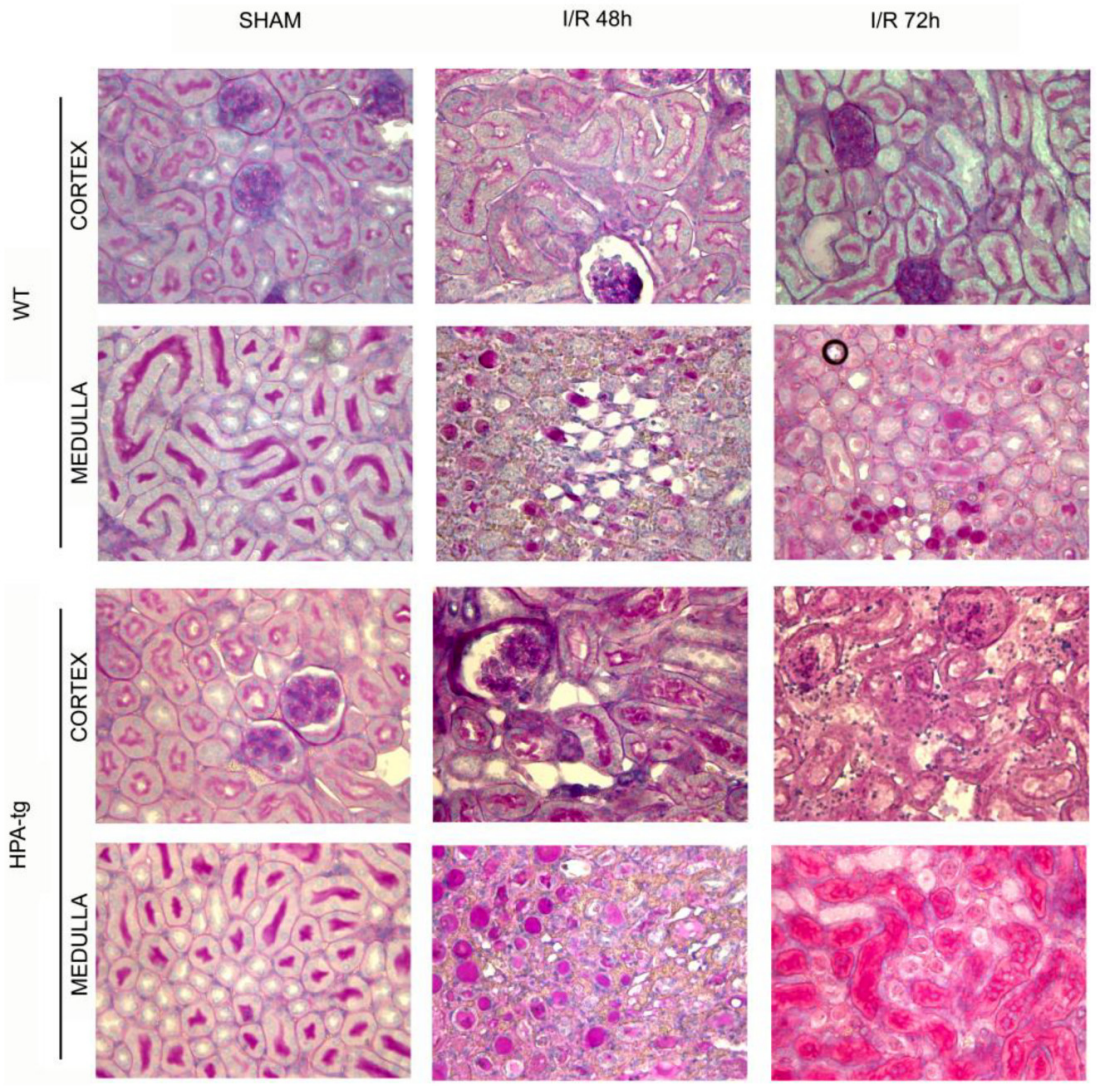
Ischemia and Reperfusion (I/R) kidney injury in wild type (wt) and HPA-tg mice. I/R kidney injury was induced in wt and HPA-tg mice by clamping of both renal arteries for 30 minutes. Mice were sacrificed after 48 or 72 hours. Shown are representative images of PAS staining of paraffin-embedded cortex and medulla sections from various experimental groups. Magnification 40X. I/R, ischemia/reperfusion.

The structural changes in the kidney tissue of control and I/R wt and HPA-tg mice were evaluated by PAS staining. At 48 h afterI/R, wt mice showed acute tubular necrosis which included tubular lysis, loss of brush border and sloughed debris in tubular lumen spaces. In wt mice the damage was partially restored after72 h (Fig 9). On the contrary, the injury in HPA-tg mice was more profound and persistent also after 72 h. In HPA-tg mice there was a significant alteration in glomeruli, and tubular structures. In particular, in HPA-tg mice I/R produced a severe tubular damage with tubular dilatation, cell detachment from basement membrane, cast formation and loss of brush border (Fig 9).

## Discussion

Activated myofibroblasts in the tubular compartment of the kidney are the main producers of collagen IV, laminin, fibronectin and are held responsible for tubular interstitial fibrosis (TIF) [14,15]. These cells have properties similar to contractile smooth muscle cells, since they express α-smooth muscle actin (α-SMA) [32]. In the kidney, myofibroblasts originate from several sources: 1) interstitial renal fibroblasts; 2) interstitial perivascular cells called pericytes; 3) fibrocytes; 4) tubular epithelial cells; and 5) endothelial cells [33,34].

Tubular epithelial-mesenchymal transition (EMT) is a process first identified in mice and, more recently, in humans, representing an important source of these cells in the kidney, and contributing to the onset of TIF and progression of chronic kidney disease (CKD) [35].

During this process, which ultimately determines renal fibrosis, the interstitial microenvironment is enriched with cytokines and chemokines released by the tubular epithelial cells in response to pathological stimuli (e.g., proteinuria, high glucose concentrations, ROS, hypoxia). Macrophages and lymphocytes attracted by chemokines release additional factors, such as transforming growth factor-β (TGF-β), epidermal growth factor (EGF), and fibroblast growth factor-2 (FGF-2) [13], all contributing to EMT of tubular epithelial cells.

Additionally, the aforementioned events are frequent in renal transplantation. In fact, as demonstrated, a significant proportion of renal transplant recipients may undergo EMT and fibrosis within a short time-period after transplantation [36]. In particular, an accelerated fibrotic process has been reported in patients undergoing delayed graft function (DGF), a slow recovery of renal function that requires dialysis treatment after transplantation [37].

DGF is a multi-factorial event influenced by several clinical factors (e.g., kidneys from non-heart-beating donor, inotropic support of the donor), donor characteristics (e.g., age, diabetes, hypertension), recipients conditions (e.g., pre-transplant dialysis treatment, number of previous transplants, allosensitization) and length of cold ischemia time [38]. The latter (ischemia/reperfusion (I/R) injury) induces local and systemic biological/cellular events, which are responsible for EMT and subsequently the development of chronic graft dysfunction [39].

Several recent findings indicate that HPSE is involved in the pathogenesis of renal fibrosis [16] in proteinuric disease such as diabetic nephropathy [20,22]. Interestingly, we have shown that this could be attributed to a pivotal leading role of HPSE in the regulation of EMT in tubular cells [24,25].

At the present time, however, no studies have defined the contribution of HPSE in H/R-associated renal injury. Only one report has shown a possible contribution of this enzyme to the onset of sepsis-induced AKI [23]. We hypothesized that HPSE is triggered in I/R renal injury and plays a role in the pathogenesis of I/R renal injury and in eliciting EMT. This could lead to the development of renal fibrosis and long-term renal dysfunction in the allograft.

In this study we have demonstrated *in vitro* and *in vivo* that HPSE is upregulated in I/R renal injury. The data also suggest that HPSE may contribute to EMT induction of tubular cells, thereby contributing to the development of organ fibrosis.

By using HK-2 cells, a human immortalized proximal tubular cell line, we provided evidence that H/R up-regulates HPSE gene expression (Fig 2A) and protein synthesis (Fig 2B). The kidney has an efficient protecting system against hypoxia and the Hypoxia-Inducible Factor (HIF) is the “Master Gene” in this system [40]. HPSE is associated with HIF-1α expression [36], which is constitutively expressed in tubular cells. In fact, HPSE silencing was shown to be associated with decreased HIF-1α level [41], suggesting that HIF-1α is downstream to HPSE. Our results confirmed that, as previously described, during H/R HIF-1a is upregulated in the hypoxic phase in tubular cells and this event could be responsible for modulation of gene expression [31] and activation of EMT via Bmi and PI3K/AKT pathway [42]. We proved that the silencing and the inhibition of heparanase prevent the accumulation of HIF-1α thus this could be a possible mechanism of EMT regulation in tubular cells

Interestingly, we observed that the HPSE inhibitor SST0001 down-regulates HPSE expression. We supposed that reduced HPSE activity upregulates the nuclear content of heparan sulphate and syndecan-1, which in turn modulates histone acetyltransferase activity [43]. However in our cellular model we observed that the treatment with SST0001 did not induce significant changes in syndecan-1 expression **(**S2 Fig) thus future studies will be necessary to explain this phenomenon.

We confirmed the results obtained in cell culture in a mouse model of I/R kidney injury. Indeed, in mice subjected to 30 minutes of renal ischemia followed by reperfusion, we detected over expression of the HPSE gene (Fig 6A) and HPSE deposition at both glomerular and tubulo-interstitial levels (Fig 6B).

The pathogenesis of I/R renal injury is very complex and includes a strong inflammatory response occurring following reperfusion due to leukocyte infiltration and activation [6]. However, the correspondence of the *in vitro* with the *in vivo* results suggests that the main trigger is hypoxia *per se*. Whether cell infiltration is involved in HPSE-dependent EMT of tubular cells requires further studies.

As expected, the EMT transcriptional program was activated after H/R (Fig 3). These modifications occurred in parallel with morphological changes in WT tubular cells. Specifically, the latter lost their typical cobblestone epithelial phenotype becoming elongated like fibroblasts.

HPSE seems to play a major role in these processes. In fact, HPSE-silenced cells and WT tubular cells treated with SST0001 maintained a normal epithelial phenotype after both hypoxia and reoxygenation, and did not initiate the EMT transcriptional program as demonstrated by stable levels of FN and α-SMA.

SST0001, a potent heparin-like inhibitor of heparanase enzyme activity, markedly decreased the ability of HPSE to release and potentiate the activity of extracellular matrix-bound FGF-2 as compared to unmodified heparin. Moreover, glycol-splitting causes heparin to lose its affinity to anti-thrombin, resulting in loss of anticoagulant activity. Collectively, the combination of high inhibition of heparanase, low release/potentiation of ECM-bound growth factors and the lack of anticoagulant activity points to N-acetylated, glycol-split heparins (e.g., SST0001) as potential anti-fibrotic, anti-angiogenic and anti-metastatic agents [44].

To the best of our knowledge this is the first evidence that HPSE is involved in H/R injury in renal tubular cells. We applied HPSE over-expressing mice presuming that in this model the biological consequences of HPSE would be magnified. As a matter of fact this hypothesis was confirmed by our experimental findings. Thus, the renal pathology in HPA-tg mice following I/R was more severe than in their wt littermates. In particular HPA-tg mice exhibited more tubular dilatation, cell detachment from basement membrane, cast formation and loss of brush border. In addition, HPA-tg mice displayed higher levels of serum creatinine following renal I/R as compared with WT mice subjected to the same procedure (Fig 8). The role of HPSE as regulator of EMT in H/R was evident also in I/R *in vivo*. Indeed I/R kidney injury induced a rapid up-regulation of mesenchymal markers (i.e., α-SMA and VIM) in renal tissues of HPA-tg mice, but not in WT animals where only a faint trend could be observed. HPSE overexpression was also associated with increased macrophages infiltration, one other pathological event that occurred in I/R [45] (S1 Fig).

It is not known if the role of HPSE in acute I/R also occurs in long term ischemia observed in CKD. If this is the case, then inhibition of HPSE may be a novel therapeutic approach to prevent CKD progression and worth further investigation. In humans, it has been shown that a significant proportion of kidney recipients display EMT features short time after organ transplantation [46], suggesting that risk factors and/or molecular events may regulate the onset of a fibrotic progression. The present data suggest that overexpression of HPSE during the ischemic events could represent a predisposing factor for activation of the EMT program. Furthermore, our *in vitro* results clearly indicate that inhibition of HPSE with SST0001 can reduce the EMT activation induced by H/R. This effect seemed more pronounced when the inhibitor was administered before hypoxia. In this perspective HPSE-inhibiting strategies could be useful both after the ischemic insult for a curative intent, but also before a “planned” ischemic event such as kidney transplantation. However, a major limitation of our study is the lack of *in vivo* testing of protective effect of SST0001 after H/R. Future studies should be undertaken to assess this objective.

Collectively, our results indicate that HPSE is a pivotal factor for the development of ischemia/reperfusion-induced EMT and its inhibition may represent a new valuable therapeutic approach to minimize or prevent fibrosis in native or transplanted kidneys.

## Supporting Information

S1 Fig Syndecan 1 (SDC-1) gene expression in tubular cells exposed to hypoxia and reoxygenation (H/R) and different SST0001 concentrations. Wild type (WT) and Heparanase-silenced (HPSE-sil) HK2 cells exposed to hypoxia and reoxygenation (H/R); WT HK-2 cells were also treated or not with 200 μg/ml SST0001. Gene expression analysis of SDC-1 evaluated by real-time PCR. Data were normalized to GAPDH expression. NORM = normoxia. Mean ± S.D (error bars) of two separate experiments performed in triplicate. **p<0.001 vs. WT CTR NORM.
(TIF)

S2 Fig Representative immunofluorescence staining for the specific macrophage-restricted F4/80 protein in renal tissue of Wild Type (WT) and HPA-tg mice subjected to Ischemia and Reperfusion (I/R). (TIF)

## References

[1] Eltzschig HK, Eckle T. Ischemia and reperfusion-from mechanism to translation. Nat Med 2011; 17: 1391–401. 10.1038/nm.2507 22064429 PMC3886192

[2] Menke J, Sollinger D, Schamberger B, Heemann U, Lutz J. The effect of ischemia/reperfusion on the kidney graft. Curr Opin Organ Transplant 2014; 19: 395–400 10.1097/MOT.0000000000000090 24905021

[3] Hotchkiss RS, Strasser A, McDunn JE, Swanson PE. Cell death. N Engl J Med 2009; 361: 1570–1583. 10.1056/NEJMra0901217 19828534 PMC3760419

[4] Eltzschig HK, Carmeliet P. Hypoxia and inflammation. N Engl J Med 2011; 364: 656–665. 10.1056/NEJMra0910283 21323543 PMC3930928

[5] Kono H, Rock KL. How dying cells alert the immune system to danger. Nat Rev Immunol 2008; 8: 279–289. 10.1038/nri2215 18340345 PMC2763408

[6] Ponticelli C. Ischaemia-reperfusion injury: a major protagonist in kidney transplantation. Nephrol Dial Transplant 2014; 29: 1134–1140. 10.1093/ndt/gft488 24335382

[7] Nankivell BJ, Borrows RJ, Fung CL, O’Connell PJ, Allen RD, Chapman JR. The natural history of chronic allograft nephropathy. N Engl J Med 2003; 349: 2326–2333. 14668458 10.1056/NEJMoa020009

[8] Bonventre JV, Zuk A. Ischemic acute renal failure: an inflammatory disease? Kidney Int 2004; 66: 480–485. 15253693 10.1111/j.1523-1755.2004.761_2.x

[9] Yang L, Humphreys BD, Bonventre JV. Pathophysiology of acute kidney injury to chronic kidney disease: maladaptive repair. Contrib Nephrol 2011; 174: 149–155. 10.1159/000329385 21921619

[10] Wynn TA. Common and unique mechanisms regulate fibrosis in various fibroproliferative diseases. J Clin Invest 2007; 117: 524–529. 17332879 10.1172/JCI31487 PMC1804380

[11] Liu Y. Epithelial to mesenchymal transition in renal fibrogenesis: pathologic significance, molecular mechanism, and therapeutic intervention. J Am Soc Nephrol 2004; 15:1–12. 14694152 10.1097/01.asn.0000106015.29070.e7

[12] Zell S, Schmitt R, Witting S, Kreipe HH, Hussein K, Becker JU. Hypoxia Induces Mesenchymal Gene Expression in Renal Tubular Epithelial Cells: An in vitro Model of Kidney Transplant Fibrosis. Nephron Extra 2013; 3: 50–58. 10.1159/000351046 23898346 PMC3711002

[13] Kanasaki K, Taduri G, Koya D. Diabetic nephropathy: the role of inflammation in fibroblast activation and kidney fibrosis. Front Endocrinol (Lausanne) 20013; 4:7. 10.3389/fendo.2013.00007 PMC3565176 23390421

[14] Carew RM, Wang B, Kantharidis P. The role of EMT in renal fibrosis. Cell Tissue Res 2012; 347: 103–116. 10.1007/s00441-011-1227-1 21845400

[15] Masola V, Zaza G, Onisto M, Lupo A, Gambaro G. Impact of heparanase on renal fibrosis. J Transl Med 2015; 13: 181 10.1186/s12967-015-0538-5 26040666 PMC4467599

[16] Vlodavsky I, Ilan N, Naggi A, Casu B. Heparanase: structure, biological functions, and inhibition by heparin-derived mimetics of heparan sulfate. Curr Pharm Des 2007; 13: 2057–2073. 17627539 10.2174/138161207781039742

[17] van den Hoven MJ, Rops AL, Vlodavsky I, et al Heparanase in glomerular diseases. Kidney Int 2007; 72: 543–538. 17519955 10.1038/sj.ki.5002337

[18] Kramer A, van den Hoven M, Rops A, Levidiotis V, Berden JH, van der Vlag J. Induction of glomerular heparanase expression in rats with adriamycin nephropathy is regulated by reactive oxygen species and the renin-angiotensin system. J Am Soc Nephrol 2006; 17: 2513–2520. 16899518 10.1681/ASN.2006020184

[19] Garsen M, Rops AL, Rabelink TJ, Berden JH, van der Vlag J. The role of heparanase and the endothelial glycocalyx in the development of proteinuria. Nephrol Dial Transplant 2014; 29: 49–55. 10.1093/ndt/gft410 24166469

[20] van den Hoven MJ, Rops AL, Bakker MA, Aten J, Rutjes N, Roestenberg P, et al Increased expression of heparanase in overt diabetic nephropathy. Kidney Int 2006; 70: 2100–2118. 17051139 10.1038/sj.ki.5001985

[21] Masola V, Gambaro G, Tibaldi E, Onisto M, Abaterusso C, Lupo A. Regulation of heparanase by albumin and advanced glycation end products in proximal tubular cells. Biochim Biophys Acta 2011; 1813: 1475–1482. 10.1016/j.bbamcr.2011.05.004 21600934

[22] Gil N, Goldberg R, Neuman T, Garsen M, Zcharia E, Rubinstein AM, et al Heparanase is essential for the development of diabetic nephropathy in mice. Diabetes 2012; 61: 208–216. 10.2337/db11-1024 22106160 PMC3237641

[23] Lygizos MI, Yang Y, Altmann CJ, Okamura K, Hernando AA, Perez MJ, et al Heparanase mediates renal dysfunction during early sepsis in mice. Physiol Rep 2013; 1: e00153 10.1002/phy2.153 24400155 PMC3871468

[24] Masola V, Gambaro G, Tibaldi E, Brunati AM, Gastaldello A, D'Angelo A, et al Heparanase and syndecan-1 interplay orchestrates fibroblast growth factor-2-induced epithelial-mesenchymal transition in renal tubular cells. J Biol Chem 2012; 287: 1478–1488. 10.1074/jbc.M111.279836 22102278 PMC3256891

[25] Masola V, Zaza G, Secchi MF, Gambaro G, Lupo A, Onisto M. Heparanase is a key player in renal fibrosis by regulating TGF-β expression and activity. Biochim Biophys Acta 2014; 1843: 2122–2128. 10.1016/j.bbamcr.2014.06.005 24937189

[26] Zaza G, Masola V, Granata S, Bellin G, Dalla Gassa A, Onisto M, et al Sulodexide alone or in combination with low doses of everolimus inhibits the hypoxia-mediated epithelial to mesenchymal transition in human renal proximal tubular cells. J Nephrol 2015; 28: 431–440. 10.1007/s40620-015-0216-y 26054821

[27] Naggi A, Casu B, Perez M, Torri G, Cassinelli G, Penco S, et al Modulation of the heparanase-inhibiting activity of heparin through selective desulfation, graded N-acetylation, and glycol splitting. J Biol Chem 2005; 280: 12103–12113. 15647251 10.1074/jbc.M414217200

[28] Pala D, Rivara S, Mor M, Milazzo FM, Roscilli G, Pavoni E et al Kinetic analysis and molecular modeling of the inhibition mechanism of roneparstat (SST0001) on human heparanase. Glycobiology. 2016 6;26(6):640–54 10.1093/glycob/cww003 26762172 PMC4847616

[29] Rivara S, Milazzo FM, Giannini G. Heparanase: a rainbow pharmacological target associated to multiple pathologies including rare diseases. Future Med Chem. 2016 4;8(6):647–80. 10.4155/fmc-2016-0012 27057774

[30] Boyango I, Barash U, Naroditsky I, Li JP, Hammond E, Ilan N, et al Heparanase cooperates with Ras to drive breast and skin tumorigenesis. Cancer Res 2014; 74: 4504–4514. 10.1158/0008-5472.CAN-13-2962 24970482 PMC4134691

[31] Conde E, Alegre L, Blanco-Sánchez I, Sáenz-Morales D, Aguado-Fraile E, Ponte B et al Hypoxia inducible factor 1-alpha (HIF-1 alpha) is induced during reperfusion after renal ischemia and is critical for proximal tubule cell survival.PLoS One. 2012;7(3):e33258 10.1371/journal.pone.0033258 22432008 PMC3303832

[32] Liu Y. Renal fibrosis: new insights into the pathogenesis and therapeutics. Kidney Int 2006; 69: 213–217. 16408108 10.1038/sj.ki.5000054

[33] Duffield JS. Cellular and molecular mechanisms in kidney fibrosis. J Clin Invest 2014; 124: 2299–2306. 10.1172/JCI72267 24892703 PMC4038570

[34] LeBleu VS, Taduri G, O'Connell J, Teng Y, Cooke VG, Woda C, et al Origin and function of myofibroblasts in kidney fibrosis. Nat Med 2013; 19: 1047–1053. 10.1038/nm.3218 23817022 PMC4067127

[35] Lan HY. Tubular epithelial-myofibroblast transdifferentiation mechanisms in proximal tubule cells. Curr Opin Nephrol Hypertens 2003; 12:25–29. 12496662 10.1097/00041552-200301000-00005

[36] Manotham K, Tanaka T, Matsumoto M, Ohse T, Inagi R, Miyata T, et al Transdifferentiation of cultured tubular cells induced by hypoxia. Kidney Int 2004; 65: 871–880. 14871406 10.1111/j.1523-1755.2004.00461.x

[37] Zaza G, Ferraro PM, Tessari G, Sandrini S, Scolari MP, Capelli I, et al Predictive model for delayed graft function based on easily available pre-renal transplant variables. Intern Emerg Med 205; 10: 135–141. 10.1007/s11739-014-1119-y 25164408

[38] Shoskes DA, Halloran PF. Delayed graft function in renal transplantation: etiology, management and long-term significance. J Urol 1996; 155: 1831–1840. 8618268 10.1016/s0022-5347(01)66023-3

[39] Kosieradzki M, Rowiński W. Ischemia/reperfusion injury in kidney transplantation: mechanisms and prevention. Transplant Proc 2008; 40: 3279–3288. 10.1016/j.transproceed.2008.10.004 19100373

[40] Nangaku M, Inagi R, Miyata T, Fujita T. Hypoxia and hypoxia-inducible factor in renal disease. Nephron Exp Nephrol 2008; 110: e1–7. 10.1159/000148256 18667839

[41] Z CZ, Luo C, Yang Z, Wang L. Heparanase participates in the growth and invasion of human U-2OS osteosarcoma cells and its close relationship with hypoxia-inducible factor-1α in osteosarcoma. Neoplasma 2010; 57: 562–571. 20845995

[42] Du R, Xia L, Ning X, Liu L, Sun W, Huang C et al Hypoxia-induced Bmi1 promotes renal tubular epithelial cell-mesenchymal transition and renal fibrosis via PI3K/Akt signal.MolBiol Cell. 2014 9 1;25(17):2650–9. 10.1091/mbc.E14-01-0044 PMC4148254 25009285

[43] Purushothaman A, Hurst DR, Pisano C, Mizumoto S, Sugahara K, Sanderson RD. Heparanase-mediated loss of nuclear syndecan-1 enhances histone acetyltransferase (HAT) activity to promote expression of genes that drive an aggressive tumor phenotype. J Biol Chem 2011; 286: 30377–30383. 10.1074/jbc.M111.254789 21757697 PMC3162396

[44] Lazo-Langner A, Goss GD, Spaans JN, Rodger MA. The effect of low-molecular-weight heparin on cancer survival. A systematic review and meta-analysis of randomized trials. J Thromb Haemost 2007; 5: 729–737. 17408406 10.1111/j.1538-7836.2007.02427.x

[45] Li L, Okusa MD. Macrophages, dendritic cells, and kidney ischemia-reperfusion injury. Semin Nephrol. 2010 5;30(3):268–77. 10.1016/j.semnephrol.2010.03.005 20620671 PMC2904394

[46] Hertig A, Verine J, Mougenot B, Jouanneau C, Ouali N, Sebe P, et al Risk factors for early epithelial to mesenchymal transition in renal grafts. Am J Transplant 2006; 6: 2937–2946. 17061992 10.1111/j.1600-6143.2006.01559.x

