# Correction: Heparanase: A Potential New Factor Involved in the Renal Epithelial Mesenchymal Transition (EMT) Induced by Ischemia/Reperfusion (I/R) Injury

**DOI:** 10.1371/journal.pone.0175618

**Published:** 2017-04-10

**Authors:** The PLOS ONE Staff

There is an error in the XML that is causing the eleventh author’s name, Samuel N. Heyman, to be indexed incorrectly as N Heyman S. The name should be indexed as Heyman SN. The publisher apologizes for the error.
