# Supplementary figures and images for: Heparanase: A Potential New Factor Involved in the Renal Epithelial Mesenchymal Transition (EMT) Induced by Ischemia/Reperfusion (I/R) Injury
Source: PLoS One. 2016 Jul 28;11(7):e0160074. doi: 10.1371/journal.pone.0160074 (PMC4965068; doi:10.1371/journal.pone.0160074)

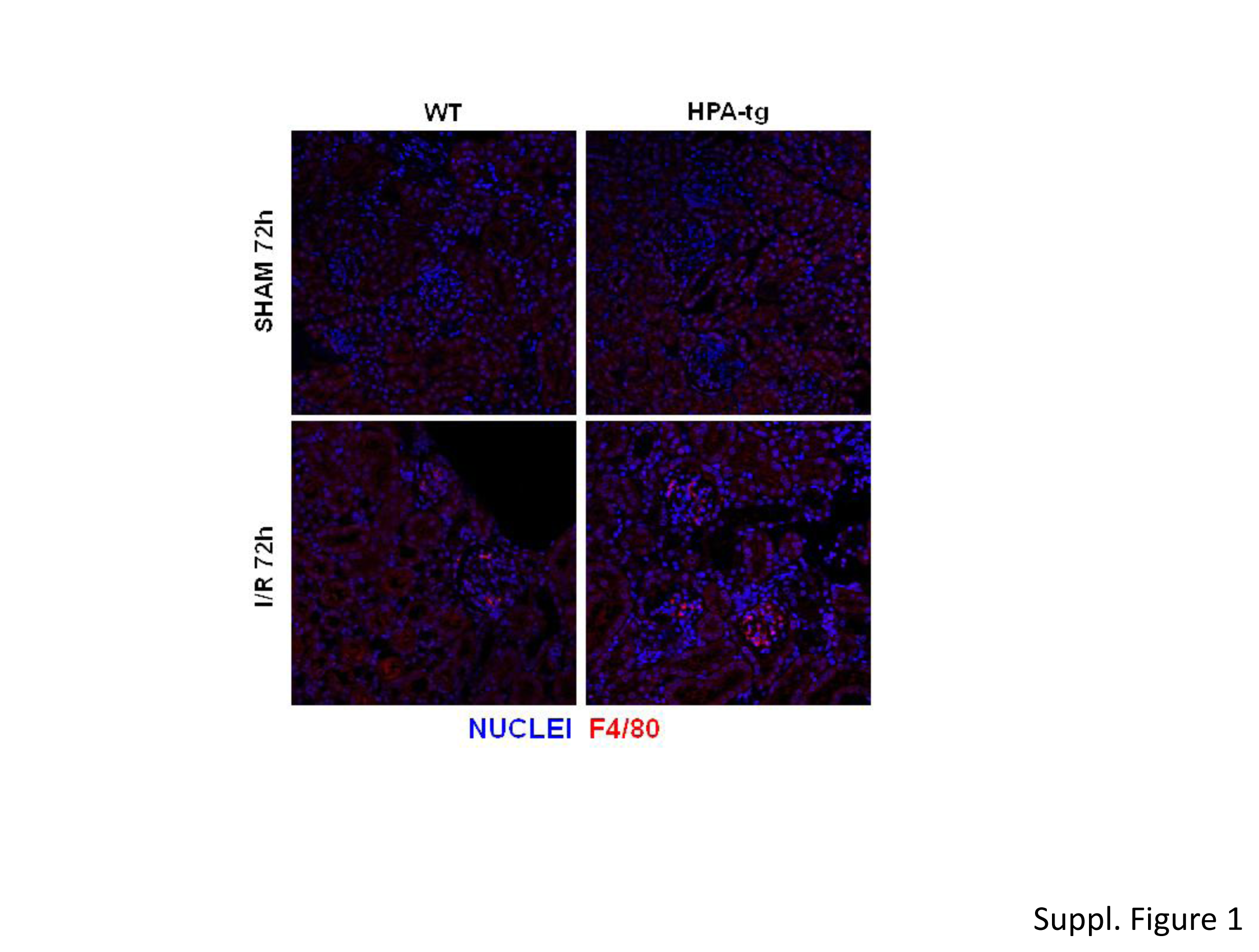

Supplement: S1 Fig — Wild type (WT) and Heparanase-silenced (HPSE-sil) HK2 cells exposed to hypoxia and reoxygenation (H/R); WT HK-2 cells were also treated or not with 200 μg/ml SST0001. Gene expression analysis of SDC-1 evaluated by real-time PCR. Data were normalized to GAPDH expression. NORM = normoxia. Mean ± S.D (error bars) of two separate experiments performed in triplicate. **p<0.001 vs. WT CTR NORM. (TIF) [file pone.0160074.s001.TIF]

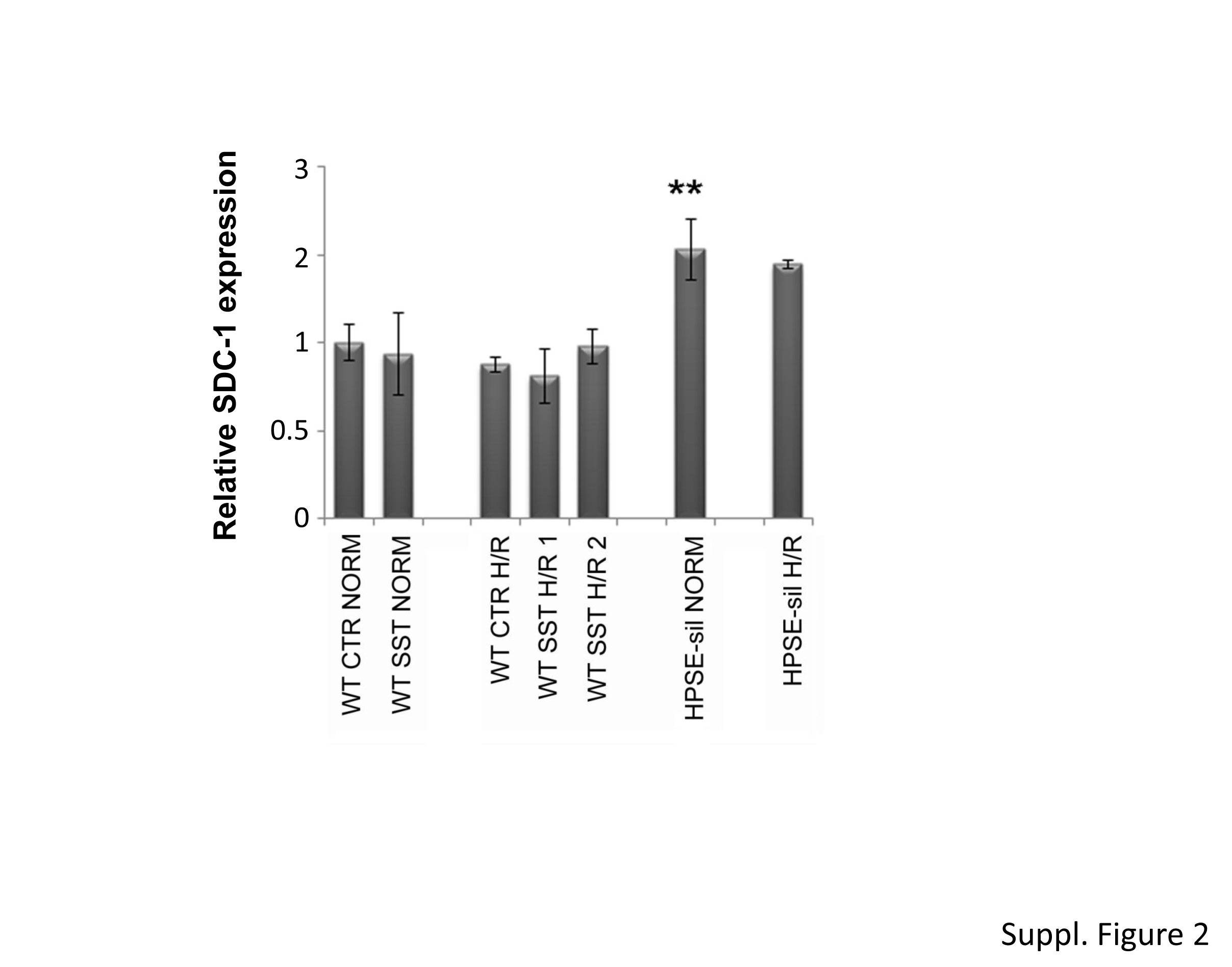

Supplement: S2 Fig — (TIF) [file pone.0160074.s002.TIF]
